# Action of Iodide of Potassium Increased by Ammonia

**Published:** 1868-12

**Authors:** 


Action of Iodide of Potassium increased by Ammonia.—It is said that the action of iodide of potassium is increased and rendered more valuable when combined with ammonia, stimulating the stomach, diffusing the blood, and with it the medicine through the system, and by chemical decomposition liberating the free iodine, and thus sending it on its salutary message.—*Humboldt Medical Archives.*

